# The Implementation of a Health Care Worker Screening Program Based on the Advanta RT-qPCR Saliva Assay in a Tertiary Care Referral Hospital in Northern Greece

**DOI:** 10.3390/life12122011

**Published:** 2022-12-02

**Authors:** Sofia Balaska, Eleftheria Parasidou, Anna Takardaki, Paraskevoula Koutra, Dimitra Chrysafi, Areti Tychala, Simeon Metallidis, Georgios Meletis, Lemonia Skoura

**Affiliations:** 1 Department of Microbiology, AHEPA University Hospital, Medical School Aristotle University of Thessaloniki, 54636 Thessaloniki, Greece; 2 First Department of Internal Medicine, Infectious Diseases Division, AHEPA University Hospital, Medical School Aristotle University of Thessaloniki, 54636 Thessaloniki, Greece

**Keywords:** COVID-19, SARS-CoV-2, health care workers, screening, saliva, RT-qPCR

## Abstract

Health care workers are at increased risk of acquiring SARS-CoV-2 infection due to different exposures in the community and in hospital settings. Interventions implemented to avoid nosocomial outbreaks include preventive testing strategies. In this report, we present results from the mass screening program applied in our hospital to all professionals, irrespective of symptoms or risk of exposure. We processed saliva specimens with real-time reverse transcription polymerase chain reaction. The total number of samples received was 43,726. Positive results were 672 and average positivity rate was 1.21%. The average positivity rate was similar to the positivity rate in the community in Greece and EU. More specifically, 80.5% of the positive participants care for patients in their daily activities, 31% experienced no symptoms before receiving the positive result, 46.1% reported a close contact with a patient or infected coworkers and 32.8% reported a close contact with infected family members. We believe that the identification of asymptomatic carriers has proved the effectiveness of the screening program by preventing the putative nosocomial spread of the virus and the depletion of workforce. In conclusion, in times of high incidence in the community, the periodic testing of health care personnel is wise and relevant for implementation costs.

## 1. Introduction

Since the emergence of novel coronavirus disease (COVID-19), health care facilities worldwide encountered tremendous challenges and were forced to adjust their infrastructures to a pandemic of massive scale. Under such circumstances, adequate staff capacity and the core function of hospitals may be hampered by health care-associated outbreaks [1].

Undoubtedly, healthcare workers (HCWs) have a higher risk of exposure to severe acute respiratory coronavirus 2 (SARS-CoV-2) due to direct patient care, thus elevating the possibility of infection compared with the general population [2]. Among them, doctors, nurses and other health care professionals who perform or assist in aerosol-generating procedures to COVID-19 patients are at higher risk. A meta-analysis of ninety-seven studies showed that the estimated prevalence of infection in medical personnel who underwent PCR testing during a screening procedure was 11% and involved mostly nurses. Most of the positive HCWs were employed in hospitalization/non-emergency wards [3].

Interestingly, the World Health Organization (WHO) revealed that the number of deaths among HCWs due to COVID-19 is largely underestimated. On the occasion of the international year of health and care workers in 2021, WHO launched a campaign to support the protection of this specific workforce [4]. As of May 2021, the number of HCW deaths related to COVID-19 that was officially reported to WHO was 6643. Performing a population-based estimation, WHO suggested that the actual number globally was 115493 until May 2021 or up to 180,000 when including deaths from high-burden countries in this estimation.

Since HCWs are at increased risk of acquiring SARS-CoV-2 infection, there is also an elevated risk of transmitting the virus to colleagues and vulnerable hospitalized patients. Most importantly, the severity of disease and mortality could be higher in older adults or patients with comorbidities [5]. Thus, COVID-19 outbreaks in hospital settings or in long-term care facilities could have dire consequences for patients or residents [6,7]. Although attack rates seem to be variable, nosocomial outbreak incidence may be as high as 60% including high mortality rates [8].

However, the actual role of HCWs in introducing or amplifying a nosocomial outbreak is not yet fully determined [8]. Paltansing and colleagues identified both HCW-to-HCW and HCW-to-patient transmission in a teaching hospital in the Netherlands. These conclusions were reached by analyzing whole-genome sequence data [9]. Likewise, another study examining four health care-associated outbreaks in a university hospital in Berlin, Germany, defined HCW-to-HCW transmission as the actual cause of the outbreaks [10].

In order to mitigate nosocomial transmission, hospitals and health care facilities worldwide applied advanced infection control interventions. The most common measures include the use of surgical masks, strict hand hygiene, adequate environmental cleansing, visitor restrictions, designated isolation and quarantine wards for confirmed or suspected COVID-19 cases and the postponement or cancellation of non-urgent surgeries. Personnel were also obliged to use standard personal protective equipment (PPE). Studies suggest that the infection of HCWs that occurred early in the pandemic reflect inappropriate use or shortages in PPE [2]. Additionally, in an effort to enhance protection of HCWs and uninfected patients, health care systems introduced different diagnostic testing policies.

Even though most screening projects applied in health care facilities concerned symptomatic rather than asymptomatic personnel [11,12], there are many studies supporting that the comprehensive screening of HCWs regardless of clinical manifestations might be beneficial. ESCMID’s official recommendations based upon CDC [13] and WHO guidance for testing [14] suggest repeated testing of HCWs two or three times per week. This procedure should be applied irrespective of symptoms or vaccination status, especially when COVID-19 incidence in the community is high and/or during nosocomial outbreaks [15]. Moreover, the testing strategies were implemented to HCWs at front line only or to all employees in health care facilities [16,17]. The screening programs involved either molecular testing methods (such as RT-PCR) or antigen-based protocols [17,18,19]. Most studies concerned the molecular detection of viral RNA from nasopharyngeal swabs (NP) [11,20,21], and limited data exist on screening testing using saliva specimens [22].

In this report, we present results from the mass screening program that was applied with HCWs of our hospital. Data collected from HCWs who tested positive during this program will hopefully give more insight into understanding the prevalence of SARS-CoV-2 carriage among HCWs and possible transmission pathways. Furthermore, we evaluated the contribution of this program as a pandemic surveillance practice applied for prevention of a potential hospital outbreak.

## 2. Materials and Methods

### 2.1. Participant Enrolment

All employees of the hospital, regardless of working exposure risk, were requested to participate in a weekly screening program for the detection of SARS-CoV-2 by RT-PCR performed in saliva specimens. The program duration was 15 months, from March 2021 to May 2022.There were no specific inclusion criteria. The essential data collected for participants were public health care coverage number, date of birth, gender and mobile number. All participants were informed about the type, the purpose and the performance specifications of the test.

Employees were instructed to collect a saliva sample for preventive testing once a week or upon symptoms. The results were delivered in maximum 24 h and the dedicated personnel directly contacted positive individuals. After completion of the project, questionnaires were handed to all participants who had received a positive PCR result. The questionnaire included inquiries about putative exposure to infected patients, close contact with a positive individual or suspected case in household settings and symptoms related to COVID-19 before positive PCR result.

### 2.2. Saliva Specimen Collection

All saliva specimens were self-collected by participants and submitted to the microbiology laboratory. Employees were advised to avoid eating, drinking, smoking, use of nasal sprays and oral hygiene products for 30 min before self-collecting the sample by spitting or drooling into an empty saliva collector tube. Specimen collection was unsupervised. Saliva specimens were stored in the laboratory at room temperature and processed within a day of collection according to the manufacturer’s instructions [23].

### 2.3. Advanta Dx SARS-CoV-2 RT-PCR Assay (Standard Biotools Inc.)

Samples were processed using the Advanta TM Dx RT-qPCR Assay, a real-time reverse transcription (RT) PCR test intended for the qualitative detection of SARS-CoV-2 nucleic acid in saliva specimens. The use of integrated fluidic circuit (IFC) arrays enables processing of 192 samples in parallel using up to 24 independent primer/probe sets. Primer/probes used were 2019-nCoV Real-Time RT-PCR diagnostic panel created by the Centers for Disease Control and Prevention (CDC). Specifically, N1 and N2 primer/probes targeting N gene enables virus detection while RNaseP primer/probe is utilized as an internal control [23].

Advanta TM Dx RT-qPCR assay enables performing PCR on saliva samples without prior RNA extraction. To replace the missing extraction step, the protocol includes a heat inactivation step at 90 °C for 10 min. Samples are also pre-diluted in phosphate-buffered saline (1X PBS) solution. Then, reverse transcription (RT) and pre-amplification using N1 and N2 primer-probe set were performed in a one-step procedure. The PCR product was again diluted and used as a template material for next step. In the next step, qPCR was set up and loaded in a nanofluidic array, using the same set of primer/probes, four times for each sample.

Along with 186 samples processed in one run, there were two sets of control samples (one set for each PCR plate) included in the run. Controls were designated as “no template” control, negative extraction control and or positive control to test the efficiency of the polymerase chain reaction and ensure that there is no contamination with external genomic material or carryover amplification material.

Raw data were collected and further analyzed in Standard Biotools Inc. (South San Francisco, CA, USA). Real-Time PCR Analysis software v.2.1, South San Francisco, U.S. and Standard Biotools Inc. Advanta Dx SARS-CoV-2 RT-PCR Assay interpretive software v1.0.1, South San Francisco, U.S. Amplification curves for each saliva sample and for control samples are available in the analysis software output. Cycle threshold (Ct) cut off limit was set at 32 cycles. Therefore, saliva samples with a Ct lower than 32 were considered positive. The limit of detection (LoD) for Advanta Dx SARS-CoV-2 RT-PCR Assay is expected to be at 6.25 GE/μL [23].

## 3. Results

The number of employees who were instructed to participate in the weekly screening testing program was 1600. The total number of saliva specimens received during the program was 43,716. Since compliance with the program varied, from March 2021 to September 2021 the number of saliva specimens admitted per week ranged from 60 to 670. From October 2021 to May 2022, the specimens received varied from 910 up to 1340 weekly. 26% out of the total specimen number were collected from males and 74% from females.

Results are summarized per month in Table 1. From the total, 5.7% of the samples received in the laboratory were rejected because they were not labeled or were unsuitable for further processing due to presence of blood, mucus or food pieces. Excluding the rejected samples, the total number of samples processed was 41,217. The number of positive PCR results was 672. Approximately 30% of the infected individuals were male and 70% were female HCWs. Out of all positive saliva specimens tested, 549 were testing positive for the first time, and 113 samples were from already confirmed positive participants re-testing. Ten specimens were classified as re-infections since the interval between infections was larger than two months. By age, 12.8% of participants with positive tests were aged 20–29 years, 17.5% were 30–39 years old, 25% were 40–49 years old, 35.7% were 50–59 years old and 9% were aged over 60 years.

**Figure 1:**
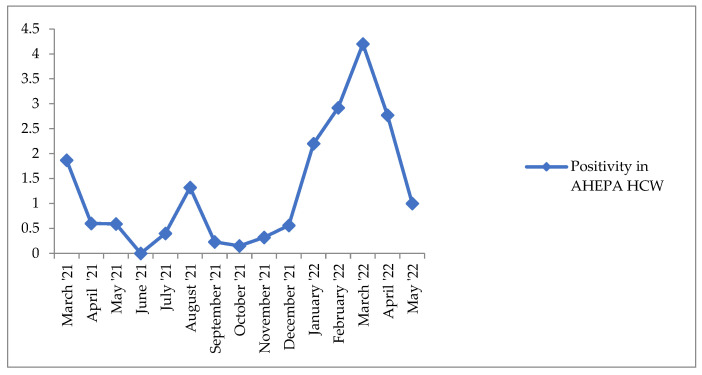
Graphical display of positivity rate % per month.

The positivity rate was calculated as the percentage of all processed COVID-19 tests that were positive and ranged from 0 to 4.20% with an average positivity rate calculated at 1.28% ± 1.25. Positivity rate per month and average positivity rate are presented in Table 2 and Figure 1. From January to April 2022, a considerable increase in the detection of positive samples was observed. For this time period, the average positivity rate was calculated to be 3.02%. The positivity rate for total samples received in this project was calculated to be 1.63%.

**Figure 2:**
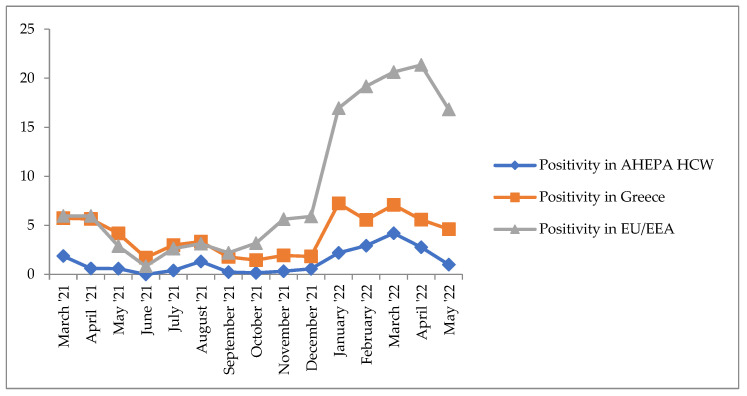
Graphical display of positivity rate % per month in AHEPA HCWs, Greece and EU/EEA.

Figure 2 displays the positivity rate among AHEPA HCWs in comparison with the average positivity rates in Greece and in the EU per month for the same timeframe. Figure 2 was created with data retrieved from ECDC weekly reports on positive cases recorded per country in EU [24].

**Figure 3:**
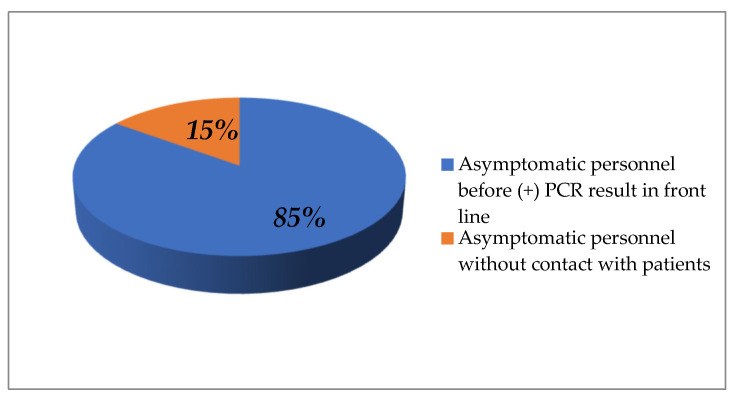
Asymptomatic personnel in front line (caring for patients).

In total, 128 questionnaires were completed from personnel who received a positive PCR result during this screening program. Results from analyzed data are presented in Table 3. In terms of patient loads, 80.5% out of the positive individuals cared for patients in their daily activities and specifically, 70.3% report that they cared for suspected or confirmed COVID-19 cases. Additionally, 32.8% reported to have close contact with a suspected or confirmed positive family member, and 46.1% reported a positive contact at work (either patient or coworker). By symptomatology, 83.6% experienced symptoms during their infection. Of notice, 31% had no symptoms before receiving the positive PCR result, and half of them remained asymptomatic during their infection. Among them, 85% reported to care for patients during their everyday practice (Figure 3).

## 4. Discussion

The implementation of a widespread testing strategy for health care personnel, irrespective of symptoms, could mount an effective control response by promptly identifying and isolating infected individuals when the viral load is high [25]. Asymptomatic personnel represent an unappreciated potential source of infection and transmission prior to the onset of symptoms has been documented [26,27].

According to our results, a considerable proportion of infected HCWs (31%) reported no symptoms before receiving the positive PCR result. Additionally, 83.6% experienced COVID-19 related symptoms after the diagnosis. Interestingly, 85% of asymptomatic HCWs care for patients in their daily activities.

Available data suggest that the percentage of infections with no symptoms ranged from 20% to 50% [28]. The infectiousness of asymptomatic carriage is either equivalent to or lower than infections with clinical manifestations [29], and almost 40% of transmission from symptomatic carriers appears prior to symptom onset [26,30,31]. Moreover, Johansson and colleagues, using a decision analytical model, estimated that more than 50% of SARS-CoV-2 new infections in the community originated from exposure to asymptomatic carriers [32].

Furthermore, Evans and colleagues, using a transition model of SARS-CoV-2 in a typical English hospital setting, calculated that nosocomial transmission could account for 20% of infections in hospitalized patients and 73% of infections in personnel [33]. Model results suggest that a regular testing policy for HCWs has a minor effect on the incidence of hospital acquired infections in inpatients but reduces the rate of HCWs’ infection by approximately 37%. It also results in very small percentages of staff depletion (equal to 0.3% per day). This percentage is considerably lower than the 20–25% absence rate that has been recorded and is due to staff sickness, staff being suspected COVID-19 cases or quarantined [33].

In the present screening project, testing was offered to all professionals regardless the level of working exposure risk. After analyzing the data retrieved from the voluntarily completed the questionnaires, 32.8% reported contact with a confirmed COVID-19 case at home and 46.1% with a coworker or confirmed COVID-19 patient. In the group, 70.3% of positive HCWs confirmed contact with COVID-19 patients during daily professional activity. Consequently, we propose that the most possible route of transmission for the majority of HCWs in this report is contacts in the hospital settings (either coworkers or patients), while a considerable percentage of participants (32.8%) was probably infected by a family member.

Published data exist supporting that the number of asymptomatic infections among health care personnel could be attributed to community infection solely [34]. However, other studies opposing to this argument claim that the risk of infection for HCWs originates from different type of exposures as well [26,35]. The CDC COVID-19 Response Team reported that 1689 individuals accounting for 11% of all confirmed cases between February and April 2020 in the US were health care personnel [36]. Among 1423 with close contacts, 55% had been in close contact with a COVID-19 patient, 27% were in close contact with an infected family member, 13% had a community-related contact and 5% had multiple contacts [36].

Other studies report high rates of SARS-CoV-2 cross- transmission between HCWs and patients or among HCWs in hospital setting [9,10]. Rücker and colleagues reported that 33% of the personnel of a hospital in Sweden were infected withSARS-CoV-2 during a health care-associated outbreak. A percentage of 96% of those positive cases belonged to front-line medical personnel caring for COVID-19 patients, while 78% of them had close contact with a contagious coworker. The study concluded that the outbreak was originated or reinforced by HCW-to-HCW transmission. On the contrary, other studies support that infection of HCWs may occur in the community, in super-spreading events or during peer-to-peer interaction outside professional activities [16].

We also found that positivity rate ranged from 0 to 4.20% among different months of the program with an average positivity rate calculated at 1.28% and an average positivity rate equal to 3.02% for the period from January to April 2022 when both number of samples admitted and number of positively detected individuals were significantly higher than in the beginning of the program. Moreover, based on official surveillance reports available from the National Public Health Organization, a considerable increase in the daily number of reported COVID-19 cases confirmed with laboratory testing from January 2022 to April 2022 was observed [37].

The positivity rate was in accordance with other published data obtained from asymptomatic screening programs or the combined screening of asymptomatic and symptomatic HCWs. In a meta-analysis of 39 heterogeneously designed studies, the proportion of positive test results ranged from 0 to 14.3% with an average calculated positivity rate equal to 1.9% for asymptomatic HCWs. This systematic search concerned studies conducted worldwide that included from 70 to 9449 participants. The authors concluded that the very high prevalence observed in some studies was most likely related to pandemic periods with an elevated incidence in certain geographical regions [18]. Other published data report positivity rates of 0.2% [38], 1.6% [20] or 3% [16] in the testing of asymptomatic health care personnel. However, the screening of symptomatic HCWs reveals positivity rates ranging from 7% to 24% [11,12,16,20,21,34].

A graphical display of average positivity rates per month in AHEPA HCWs, Greece and EU/EEA reveals that the point prevalence in HCWs is similar to the prevalence in the general population in Greece and EU, though a magnitude lower. This observation contradicts other studies that propose a disproportionate rate of infection in health care personnel than in general population [26,39,40].

Apart from the implementation of periodic testing, the reduction of SARS-CoV-2 transmission is also believed to depend on method sensitivity and the timeline of result reporting [26]. The Advanta RT-qPCR Assay assigned for SARS-CoV-2 detection in our hospital is a molecular method with similar specificity and sensitivity when saliva samples and paired nasopharyngeal samples were tested in parallel [41]. The time necessary for the completion of the protocol since the receipt of samples in the laboratory is about seven hours, and the turnaround time for a result is 7 to 24 h. A limitation to fast result reporting is the time required to gather the necessary number of samples that are processed simultaneously to conduct the assay (186 samples).

Saliva appears to be an appealing alternative biomaterial in RT-PCR testing for the identification of SARS-CoV-2. Saliva collection is an easy, non-invasive, painless procedure that needs little instruction and does not necessarily require clinician supervision. Therefore, saliva represents an ideal material for self-sampling at home or at work [42]. Unlike nasopharyngeal swabs, it is a cost-effective diagnostic fluid since there is no need for special tubing or transport media to collect saliva. In addition, there are several protocols that exclude the RNA extraction step prior to RT-PCR, thus further reducing diagnosis time [41,43]. However, in the present report, a percentage of 5.7% of received saliva in the laboratory could not be further processed because these were unsuitable or unlabeled.

In the present study, limitations include the absence of clinical information and limited data gathered about the types of exposure for the infected personnel. Data obtained from the analysis of questionnaires represent only 19% of positive results due to limited compliance with the survey. Furthermore, data from questionnaires rely on personal declarations and could be considered relatively biased since they are not supported by any scientific reports. Saliva samples were stored at room temperature until processed for about 24 h, and since RNA viruses are targets for RNAses, this fact could have probably influenced the quality of RNA in the samples to some extent. However, the procedure was in accordance with the manufacturer’s instructions for use [23], and previous evaluation of the assay proves its diagnostic accuracy [41].

Data obtained from this program indicate that regular testing helped with identifying and isolating a considerable number of asymptomatic personnel and hopefully prevented the spread of the virus in inpatients and health care workers. An additional advantage is that systematic testing maintains work productivity and avoids unnecessary workforce depletion, also highlighted in other studies [33,44]. Concerning the transmission of the virus to HCWs, we observed different types of exposures, though case incidence among HCWs was not higher than expected by community infection.

We conclude that in times of low incidence in the general population and assuming that the majority of a health care workforce has been fully vaccinated and conforming to related infection control interventions, there should be a strict cost–benefit consideration in the implementation of surveillance screening programs.

## Figures and Tables

**Table 1:** Total results of samples processed to the laboratory per month.

Saliva Samples/Month	March 2021	April 2021	May 2021	June 2021	July 2021	August 2021	September 2021	October 2021	November 2021	December 2021	January 2022	February 2022	March 2022	April 2022	May 2022	Total
Negative	367	2611	1344	369	252	450	1292	3401	4966	4643	4699	4555	4765	3373	3468	40,555
Positive	7	16	8	0	1	6	3	5	16	26	107	137	209	96	35	672
Total	374	2627	1352	369	253	456	1295	3406	4982	4669	4806	4692	4974	3469	3493	41,217

**Table 2:** Positivity rate %.

Saliva Samples/Month	March 2021	April 2021	May 2021	June 2021	July 2021	August 2021	September 2021	October 2021	November 2021	December 2021	January 2022	February 2022	March 2022	April 2022	May 2022	Average
Positivity Rate %	1.87	0.6	0.59	0	0.4	1.32	0.23	0.15	0.32	0.56	2.20	2.92	4.20	2.77	1.00	1.28

**Table 3:** Total results from questionnaires received.

Reply	Front Line Personnel	Contact with Suspected or Positive Patient	Related COVID-19 Symptoms	Symptoms before Positive PCR Result	Contact with Positive Family Member	Contact with Positive Person at Work
Yes	103 (80.5%)	90 (70.3%)	107 (83.6%)	88 (69%)	42 (32.8%)	59 (46.1%)
No	25 (19.5%)	38 (29.7%)	21 (16.4%)	40 (31%)	86 (67.2%)	65 (52.4%)
Total	128	128	128	128	128	124

## Data Availability

Data are included in Table 1 and Table 3 of the article.
